# Assessment of Mandibular Canal Using Cone-Beam Computed Tomography (CBCT) and Its Relevance in Post-Operative Neurosensory Disturbances Following Bilateral Sagittal Split Osteotomy Setback

**DOI:** 10.7759/cureus.36004

**Published:** 2023-03-10

**Authors:** Sofiya Kanneth, Sankar Vinod V, Varghese Mani, Arun George, Atic Thomas

**Affiliations:** 1 Department of Oral and Maxillofacial Surgery, Mar Baselios Dental College, Kothamangalam, IND

**Keywords:** neurosensory disturbances, cbct, inferior alveolar nerve, bsso, bilateral sagittal split osteotomy

## Abstract

Aim: Damage to the inferior alveolar nerve (IAN) during bilateral sagittal split osteotomy (BSSO) causes neurosensory disturbances (NSD) of the lower lip and chin. The study aims to investigate the pre-operative position and course of the mandibular canal using cone-beam computed tomography (CBCT) and orthopantomogram (OPG) and compare if there is any difference in NSD following the BSSO setback procedure.

Materials and methods: This is an observational study. This study was conducted in the Department of Oral and Maxillofacial Surgery, Mar Baselios Dental College, Kothamangalam, from November 2017 to October 2019. Thirty patients undergoing BSSO setbacks are selected based on the inclusion and exclusion criteria and randomly grouped into two groups - group A (15) and study group B (15). OPG was done for both groups as it was required during surgical orthodontic evaluation and preoperative assessment of the mandibular canal was done using CBCT in group B. Neurosensory assessment was done in all the patients and compared the results.

Results: NSD were reported in all the patients on the first postoperative day. The correlation between subjective and objective evaluation showed absolute coincidence but nearly all the patients returned to their presurgical situation within six months in group B and one year in group A who had NSD problems.

Conclusion: From the study, it can be concluded that a CBCT scan is a useful and reliable modality in the pre-operative evaluation of the mandibular canal in patients undergoing BSSO setback procedures, which helps to improve the patient’s post-operative care and quality of life.

## Introduction

Bilateral sagittal split osteotomy (BSSO) is the most stereotypical orthognathic surgical treatment for jaw deformity correction [1]. The statement mentioned is unique to this article. While most of the reference articles state that BSSO is one of the ‘most common’, ‘standard procedure’, for the treatment of mandibular deformities no article uses the word ‘stereotypical’ in their text. Although, the literature review does show that BSSO is one of the most widely used and preferred surgical procedures in the treatment of mandibular deformities and hence the statement mentioned in the article seems to agree with the already published articles. The ramus and posterior body of the mandible are divided sagitally in this method, allowing for either setback or advancement. The inferior alveolar nerve (IAN) is at high risk of damage during BSSO with respect to the placement and course of the mandibular canal [1]. Numerous prospective and retrospective investigations show that neurosensory disruption (NSD) occurs 80% to 100% of the time following BSSO [2]. Expertise in the anatomic position and course of the mandibular canal is essential to reducing damage to the IAN during BSSO. Anatomic investigations of the mandibular canal have been conducted, however, they have mostly used human cadaver mandibles [3,4]. Many factors are thought to be responsible for the development of neurosensory disturbances after BSSO, as well as surgical procedures, the extent of mandibular movement, patient age, fixation methods, the type of screws used, surgeon experience, and the timing of postoperative neurosensory evaluation [5]. The literature indicates that planned surgical incision can reduce postoperative edema, pain, speech and chewing problems, and neurosensory disturbances. It also improves patient satisfaction [6].

In our study, cone-beam computed tomography (CBCT) and orthopantomogram (OPG) were used to establish the pre-operative anatomical position of the mandibular canal and the course of the mandibular canal. We also wanted to compare if there is any difference in neurosensory disturbances following the BSSO setback procedure.

## Materials and methods

This is an observational study. The Institutional Ethical Review Board provided the necessary approval and its reference number was IEC/03/OMFS/MBDC/2017. The sample size was estimated using the formula NA = 2σ2 (z1−α/2+zβ)2/d² where d is the effective size calculated from a pilot study. This study was conducted in the Department of Oral and Maxillofacial Surgery, Mar Baselios Dental College, Kothamangalam, from November 2017 to October 2019. After meeting the following inclusion and exclusion criteria, 30 patients between the ages of 18 and 30 were chosen at random and split into two groups (15 patients in each group). The procedures were explained and an informed consent form was obtained. Inclusion criteria include (1) Radiographically completely bilaterally corticated IAN canal; (2) Age group of patients ≥ 18 years - 30 years; (3) Patients undergoing BSSO setback procedure. Exclusion criteria include (1) Class III dental malocclusion alone; (2) Patient with a previous history of neurosensory disturbance; (3) Presence of developmental disorders, pathology, trauma, or previous treatment that could affect mandibular canal position; (4) Patients with any systemic problems; (5) Skeletal mandibular deviation or a structural defect in the vertical plane.

**Figure 1:**
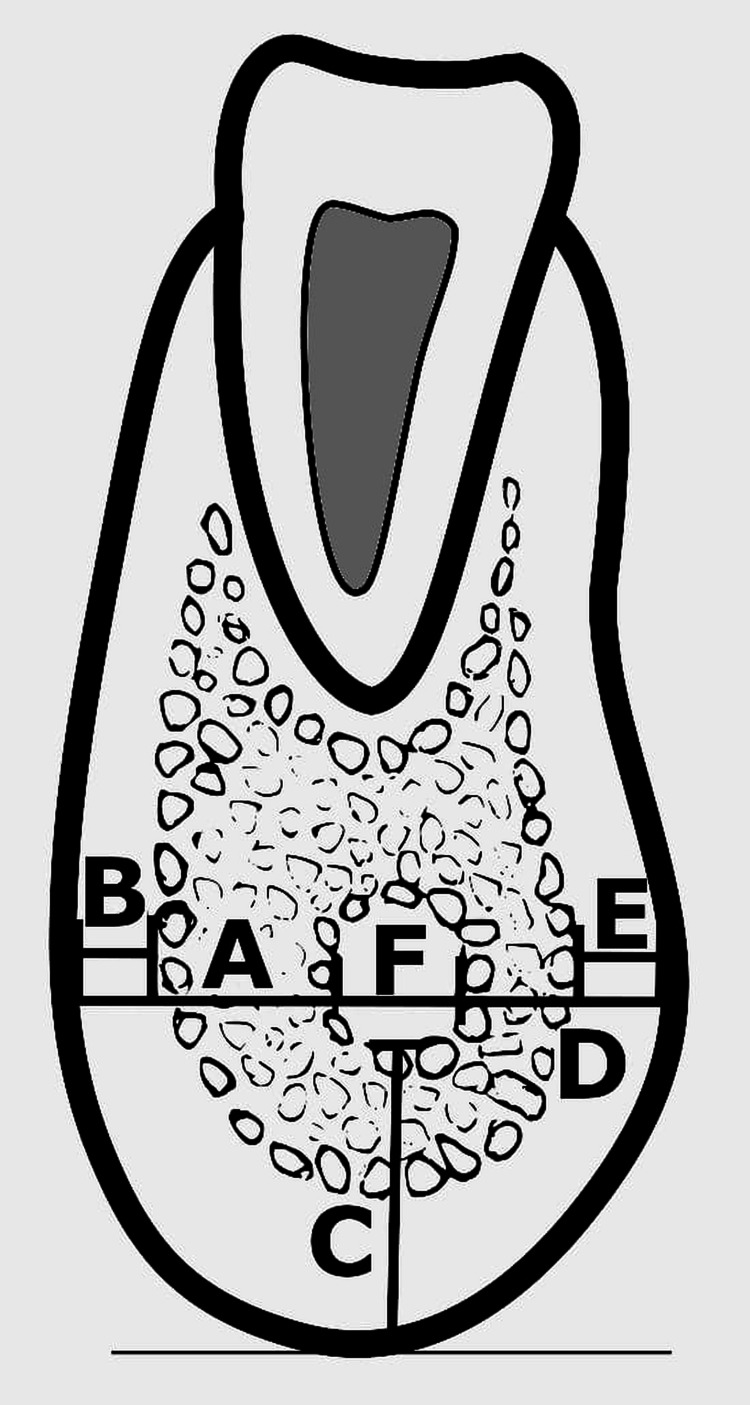
Coronal section of mandible indicating horizontal and vertical measurements in CBCT A is the distance from the canal to the buccal cortex of the mandible. B is the thickness of the buccal cortical plate. C is the distance from the canal to the lower border of the mandible. D is the distance from the canal to the lingual cortex of the mandible. E is the thickness of the lingual cortical plate. F is the diameter of the canal. CBCT: cone-beam computed tomography

**Figure 2:**
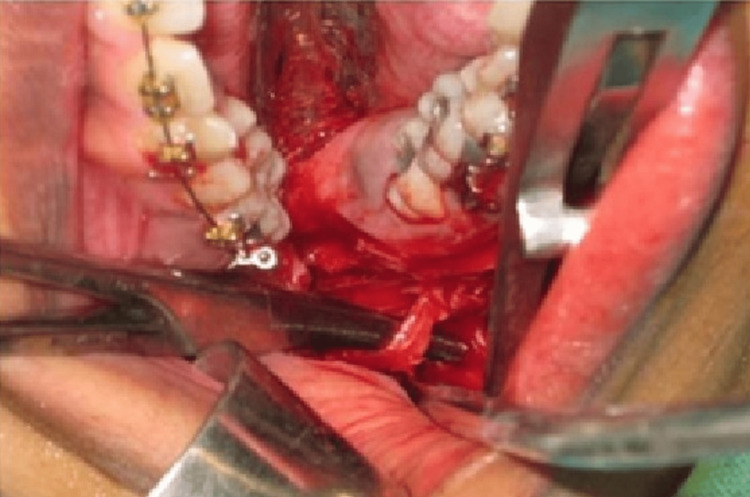
Sagittal split with the neurovascular bundle on the distal segment

**Figure 3:**
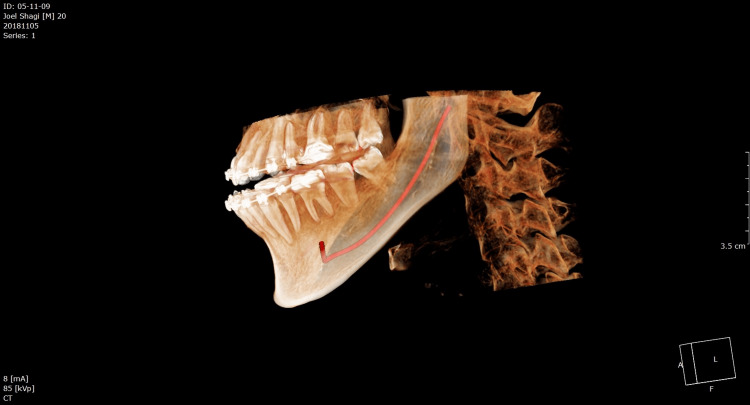
CBCT imaging of the post-operative BSSO setback CBCT: cone-beam computed tomography, BSSO: bilateral sagittal split osteotomy

In subjects in the control group (A), surgery was performed after assessment of IAN position using panoramic radiographs, whereas, in the study group (B), both panoramic radiographs and a CBCT were obtained before surgery. Cephalometric analysis for orthognathic surgery (COGS) analysis was performed for all patients. Only one team worked, saline was used for irrigation, and the painkiller as tablet Zerodol was prescribed two times a day for five days which was given to all patients. Amoxicillin 500 mg three times a day for eight days was prescribed for all patients. A soft diet with limited movements was advised and the recovery was monitored for one month for any complications. The average time for the surgery was kept at a minimum of about 1 hour to 1.5 hours so that fewer complications would happen. Bone sections were made based on linear measurements obtained preoperatively with CBCT in group B, whereas in group A all bone sections were made based on average values from the literature. All surgical processes were carried out by the same skilled surgical team. All the pre- and post-surgical orthodontic management also were done by the same team. Preoperative and postoperative neurosensory disturbances were assessed by the same team in both groups at week one preoperatively and postoperatively at week one, month one, month three, and month six. The CBCT device used in this study was the NewTom GiANO model (NewTom, Giano, Verona, Italy). The reconstructed image has a slice thickness of 0.15 mm and a field of view (FOV) of 11 x 8 cm. Panoramic, axial, coronal, and sagittal sections were obtained using NNT Viewer version 2.21 (Quantitative Radiology, Verona, Italy) imaging software to localize the canal. Cross-sectional and panoramic sections were used to mark the IAN from the first visible location of the lingual to the mental foramen on both sides (Figures 1, 2, 3).

The buccolingual diameter of the inferior alveolar canal and the thickness of the cortical plate were calculated bilaterally at three different positions. (1) Position 1: Between the first and second molar (P1), (2) Position 2: Between the second and third molar (P2), (3) Position 3: Distal third molar (P3). Measurements were taken with a digitized ruler at each position.

(A) is the distance from the canal to the buccal cortex of the mandible, (B) is the thickness of the buccal cortical plate, (C) is the distance from the canal to the lower border of the mandible, (D) is the distance from the canal to the lingual cortex of the mandible, (E) is the thickness of the lingual cortical plate, and (F) is the diameter of the canal.

Neurosensory evaluation

The pre-operative neurosensory disturbance was checked with subjective and objective methods one week prior to the surgery. All the patients were seated in the dental chair in a relaxed and comfortable position in a silent room, relaxed lips and eyes closed. Four zones were marked over the chin and lip. The lower two zones represent the regions innervated by a mental branch of the IAN, and the upper two zones represent the regions innervated by the labial branch.

Subjective examination

A questionnaire was prepared to assess the alteration in the lip and mental region and was given to the patient. A 5-point scale was used to describe the sensation: 1. No sensation. 2. Almost no sensation. 3. Some sensation. 4. Almost normal sensation. 5. Completely normal sensation.

Objective examination

I. Mechanoceptive Tests

A. Light touch: (a) Static light touch: A wisp of cotton lightly touched over the skin to assess the integrity of myelinated afferent A-beta axons. Four zones were assessed individually. The presence of sensation was noted based on the positive or negative reply. (b) Brush directional stroke: A small paintbrush was moved from the left side to the right side and vice versa on each zone which is used to assess the integrity of A-Beta myelinated axons.

B. Static two-point discrimination: The cutaneous surface of each region was tested with a sharp metallic compass. The procedure was started with the two points closed and progressively opening the edges to 1 mm increments until the patient was able to differentiate between two points of contact. The sharp points help to analyze unmyelinated C fibers and small myelinated A-delta fibers, the blunt points help to assess large myelinated A-alpha fibers. Two millimeters more than the preoperative measurement was deemed to be a normal distance.

II. Nociceptive Tests

1. Pin pressure nociception: With needles made specifically for this investigation and weighing between 0.5 and 15 grams, each zone was measured. The needles were hung on a metallic loop and allowed to touch the zones by their weight. The smallest needle that the patient perceived as being sharp was recorded. If a 1 gm difference was present from the preoperative value, the result was estimated as abnormal. The test is used to assess small myelinated A-delta and C fibers that convey painful stimuli.

2. Thermal discrimination test: The test is used to assess unmyelinated and small myelinated fibers. Cold sensation to C-fibers and warm to A-Delta fibers.

a. Hot test: A test tube with hot (50°C) water is placed on the testing area. Positive or negative response regarding the sensation is noted.

b. Cold test: Using a test tube with cold water at 15°C placed at the selected zone. A-Delta fibers transmit stimuli encoded for temperature and pain. Ask the patient to respond if he/she is feeling any kind of sensation.

Surgical procedure

All BSSO procedures were carried out using the Obwegeser-Dal Pont approach with the Hunsuck and Epker modification. With a hematoxylin pencil, the measured CBCT values were indicated approximately between the first and second molars, the second and third molars, and distal to the third molar after the incision. The anterior ramus was held in place with curved Kocher forceps. A 701 fissure bur was used to indicate the medial ramus osteotomy, which was inserted slightly above the lingula and parallel to the occlusal plane. The position of the IAN in its intraosseous bed was compared to the preoperative CBCT imaging (Figure 2). It was gently removed if it was attached to the proximal section. In both groups, the IAN was exposed with the blunt end of the periosteal elevator if it was still encased in the mandibular canal. A total of 0.9% normal Saline and 10% Betadine were used for irrigation. The patient was placed in maxillomandibular fixation (MMF) using a custom acrylic splint and a 0.26 mm stainless steel ligature wire, which fixed the mandible in its normal occlusal relationship to the maxilla. Excess bone was removed from the proximal segment to achieve mandibular setback. Fixation was achieved using a buccally placed 2 mm four-hole mini plate with a gap and 2 x 6 mm four monocortical titanium screws (Figure 3). The MMF and the plastic splint were removed after satisfactory fixation. The wounds were closed with absorbable 3-0 Vicryl suture material after hemostasis was achieved.

The average duration of the surgical procedure was 1 hour to 1.5 hours. The post-operative medications were the same for all the patients. They are Cefotaxime 200 mg orally 12 hours for five days, Metronidazole 400 mg orally 8 hours for five days, Diclofenac 50 mg + Serratiopeptidase 10 mg orally 8 hours for five days, Pantoprazole 40 mg once daily orally half an hour before food daily morning, Multivitamin supplements once daily orally for two weeks, 0.2% Chlorhexidine gluconate mouth rinse - 15 ml rinse after meals.

All patients were instructed to maintain good oral hygiene, a strict soft diet, steam inhalation, and warm saline gargle. The recovery period was between six months to one year after the surgery for all the patients.

All data were gathered and uploaded from the data collecting form to a spreadsheet (Microsoft Office Excel 2010), where it was statistically evaluated using Statistical Product and Service Solutions (SPSS) (IBM SPSS Statistics for Windows, Armonk, NY) software. The independent samples test and the chi-square test were used to test differences between groups statistically, with significance set at P 0.05.

## Results

Thirty patients undergoing BSSO setbacks are selected based on the inclusion and exclusion criteria and randomly grouped into two groups (15 subjects in each group). In this study, 15 patients were included, with ages ranging from 18 to 30. In group A, there were eight females and seven males, and in group B, there were five females and ten males.

Morphology of mandibular canal

The mean distance between the mandibular canal and the buccal cortex was 6 mm (point A). Position P2 had the shortest distance between the canal and the inferior border (point C). The mean distances of positions P1 and P3 were almost the same ((SD) P1 right 1.93, left 2.41, P3 right 1.27, left 1.52), suggesting that the inferior alveolar canal is slightly higher in the third molar region. The mean distance from the lingual margin of the mandible (point C) to the canal was compared, and the distance was greater at the P1 position (SD right 0.85, left 1.26) and lesser at positions P2 and P3. This suggests that in the analyzed sample, the mandibular canal increases somewhat toward the mental foramen.

Subjective examination

Subjective examination revealed a 53% maximum of sensation in the control group and a 73% maximum in a study group at postoperative month 6 (Tables 1, 2, 3).

**Table 1:** Comparison of CBCT measurements obtained at positions P1, P2, and P3 on the right and left side (A) is the distance from the canal to the buccal cortex of the mandible, (B) is the thickness of the buccal cortical plate, (C) is the distance from the canal to the lower border of the mandible, (D) is the distance from the canal to the lingual cortex of the mandible, (E) is the thickness of the lingual cortical plate, and (F) is the diameter of the canal. Position 1: Between the first and second molar (P1) Position 2: Between the second and third molar (P2) Position 3: Distal third molar (P3) CBCT: cone-beam computed tomography

	P1		P2		P3	
	Left	Right	Left	Right	Left	Right
A	6.4527	6.1293	6.8593	6.824	5.1313	5.23
B	2.552	2.8173	2.53	2.456	2.1287	2.3833
C	7.0807	6.9573	5.5313	6.1967	7.14	7.3153
D	3.236	3.2053	2.592	2.132	3.4993	2.5893
E	1.6573	1.9367	1.6507	1.496	1.7047	1.7913
F	2.3033	2.4367	2.264	2.6213	2.4587	2.634

**Table 2:** Comparison of objective examination of both groups at post-operative three month

Test	Area	Side	Group A	Group B	Chi-square Value	P value
Brush directional stroke	Lip	Right	5	11	4.8214	0.028108
		Left	6	10	2.1429	0.143235
	Chin	Right	6	9	1.2	0.273322
		Left	7	10	1.2217	0.269023
Light touch examination	Lip	Right	5	11	4.8214	0.028108
		Left	4	10	4.8214	0.028108
	Chin	Right	5	9	2.1429	0.143235
		Left	5	10	3.3333	0.067889
Pin pressure examination	Lip	Right	5	10	3.3333	0.067889
		Left	4	11	6.5333	0.010587
	Chin	Right	5	10	3.3333	0.067889
		Left	4	11	6.5333	0.010587
Static two-point discrimination	Lip	Right	5	8	1.2217	0.269023
		Left	5	7	0.5556	0.456057
	Chin	Right	5	10	3.3333	0.067889
		Left	5	10	3.3333	0.067889
Thermal discrimination test (Cold)	Lip	Right	4	9	3.3937	0.065447
		Left	5	10	3.3333	0.067889
	Chin	Right	5	12	6.6516	0.009907
		Left	4	11	6.5333	0.010587
Thermal discrimination test (Hot)	Lip	Right	4	9	3.3937	0.065447
		Left	5	10	3.3333	0.067889
	Chin	Right	5	12	0.136037	0.009907
		Left	4	11	6.5333	0.010587

**Table 3:** Comparison of objective examination of both groups at post-operative six month

Test	Area	Side	Group A	Group B	Chi-square Value	P value
Brush directional stroke	Lip	Right	7	13	5.4	0.020137
		Left	7	12	5.4	0.020137
	Chin	Right	7	14	7.7778	0.005289
		Left	7	13	5.4	0.020137
Light touch examination	Lip	Right	6	13	7.0335	0.008
		Left	5	12	6.6516	0.009907
	Chin	Right	7	14	7.7778	0.005289
		Left	6	13	7.0335	0.008
Pin pressure examination	Lip	Right	6	13	7.0335	0.008
		Left	5	13	8.8889	8.8889
	Chin	Right	6	13	7.0335	8.8889
		Left	5	13	8.8889	0.002869
Static two-point discrimination	Lip	Right	5	11	4.8214	0.028108
		Left	5	11	4.8214	0.028108
	Chin	Right	5	12	6.6516	0.009907
		Left	5	13	6.6516	0.009907
Thermal discrimination test (Cold)	Lip	Right	6	13	7.0335	0.008
		Left	5	12	6.6516	0.009907
	Chin	Right	5	13	8.8889	0.002869
		Left	5	13	8.8889	0.002869
Thermal discrimination test (Hot)	Lip	Right	5	13	8.8889	0.002869
		Left	5	12	6.6516	0.009907
	Chin	Right	5	13	8.8889	0.002869
		Left	5	13	8.8889	0.002869

The majority of patients (30-40%) had only some sensation (scale 3) on subjective examination, which was considered an abnormal sensation.

Objective examination

In the present study, all tests for lip and chin were performed separately and the results of the two groups A and B were compared. Light touch and directional brush stroke observation tests, pin pressure nociception, two-point static discrimination, and thermal tests also showed some response (scale 3) on the first postoperative day. That is, practically all of the patients in this study exhibited a bilateral neurosensory loss on the first postoperative day.

Our study showed progressive improvement in sensation from the first postoperative week to six months (group A: 80-93%, group B: 100%) in both groups. The results showed higher sensation in group B. A significant result was obtained from three months postoperatively for lip and chin. In all patients, the neurosensory response to the preoperative situation had to be followed up for one year.

## Discussion

Neurosensory disorders are among BSSO's most severe side effects [7]. Previous studies have identified a number of risk factors for medial periosteal dissection injuries, including fixation techniques, postoperative edema or bleeding, patient age, the osteotomy line, and the direction of mandibular movement. The postoperative neurosensory disturbances are compared here with a group (A) who had panoramic radiographs taken prior to surgery and group B who had CBCT. Literature reviews say that the planned surgical cut may reduce post-operative neurosensory disturbances, which can improve the quality of care.

The present study used CBCT in one group of subjects (the study group) and panoramic radiographs in the other group (the control group). Yu IH et al. conducted a study using 3D CT prior to the BSSO [5]. Whereas Yoshioka I et al. [8], and Verweij JP et al. [9] have reported that 2D radiography gives reproducible measurements and is used to find morphological features of the mandibular region. In this study, CBCT was used to locate the inferior alveolar canal prior to surgery since it has reduced radiation dosage. Because the CBCT scanning machine is smaller and less expensive, it may be used efficiently in a dental clinic for less money. A study by Meyer MK [10] found that CBCT scans can be an important part of a surgeon's preoperative assessment, particularly in determining the buccolingual and inferior position of the canal. The use of CBCT can assist in the planning of surgical osteotomies.

There are differing opinions on the removal of third molars before BSSO. Some studies have suggested that removing third molars before surgery may help prevent intraoperative complications, such as bad splits and injury to the IAN [11,12]. Other authors have recommended removing third molars at the same time as orthognathic surgery, citing fewer postoperative complications, such as hypoaesthesia, with this approach [13]. In this study patients with mandibular third molars that caused potential impingement of the IAN had the molars removed at least 9-12 months prior to surgery, and cases of post-surgical neurosensory dysfunction (NSD) were excluded from the study. However, a study by Doganay O et al. [14] found that the concurrent extraction of third molars did not affect neurosensory outcomes.

In the present study, the canal to buccal cortical thickness was 6 mm on average. The increased buccal cortical thickness helped to reduce the damage to the canal during the surgery. The postoperative results showed improved sensation. According to the study, Rajchel J et al. [15] and Sekerci AE et al. [16] reported the distance between the cortical plate and the mandibular canal was highest between the second and first molars, and it decreased in the third molar area.

In this study, the average distance from the inferior margin of the mandible to the mandibular canal (Point C) was compared with the right and left sides at three different sites and showed the least distance at the P2 position than at other positions. The positions of P1 and P2 mean distances were almost equal. This gives the impression that the mandibular canal is placed slightly higher in the third molar region. The neurovascular bundle, according to Gowgiel JM et al. [17], is located in the premolar region of the mandible, around 10 mm below the lower border and quite close to the inferior border. In the present study, the mean distance from the lingual margin of the mandible to the canal (Point D) was compared with the right and left sides at three different sites. The distance was longer at the P1 position. The distance from the lingual margin of the mandible to the canal was less in the P2 and P3 positions. In the analyzed sample size, this shows that the mandibular canal is descending slightly toward the mental foramen. In the studies by Gowgiel JM et al. [17] and Aizenbud D et al. [18], it was found that the bundle of nerves and blood vessels from the mandibular foramen to the mental foramen was in good shape.

In this study, the vertical and horizontal position of the lingula was not taken as a parameter due to the limited surgical field of vision and the search for the anti-lingula. The neurovascular bundle may have been compressed and stretched severely, yet the horizontal cut at the posterior region was made above the lingula. The postoperative results showed improved sensation when compared with the control group. The subjective examination showed 53% maximum sensation with the control (group A) and 73% maximum with the study group (group B) at six months postoperatively. In their study, Politis C et al. [19] used a modified buccal osteotomy to reduce nerve damage. As a result, NSD changes were reported to go from 15.12% to 9.4%. In this study, we used blunt chisels to reduce nerve damage while splitting Nakagawa K et al. [7] said that the process of splitting is related to the development of IAN neurosensory impairment (NSI).

Teerijoki-Oksa T et al. [20] and Fridrich KL et al. [4] reported that neurapraxia and axonotmesis often result in spontaneous recovery, which occurs most frequently within six months of surgery, regardless of the patient's age. However, August M et al. [3], and Verweij JP et al. [9] found that age may be a significant factor in recovery from nerve damage. They found that older patients (above 30 years) may be more prone to iatrogenic nerve damage during surgery due to axonal atrophy and degeneration. Verweij JP et al. [21] also reported a lower probability of recovery and a longer time to recover in older patients. However, in this study, all the patients were aged 18-30 years, so it was not possible to compare the results of different age groups.

In this study, we used both subjective and objective methods. All the tests were done for the lip and chin separately and the results were compared between groups A and B. The light touch observation and brush directional stroke tests showed almost similar results on the first postoperative day. The majority of the patients (30-40%) had only some sensation on the subjective examination, which was considered an abnormal sensation. But pin pressure nociception, static two-point discrimination, and thermal tests had some response on the first postoperative day itself. The study conducted by Antony PG et al. [22] found similar results. They found that 85% had no response to the above tests. Our study showed a progressive improvement in the sensation from the postoperative first week to six months in both groups. The results showed a higher sensation in group B. The significant results were obtained at postoperative three months for the lip and at postoperative six months for the chin. Teerijoki-Oksa T et al. [20], Roychoudhury S et al. [23], and Antony PG et al. [22] reported that recovery of nerve function markedly occurs in the first three to six months.

In the present study, almost all of the patients in this study showed a bilateral neurosensory deficit on the first postoperative day. All of the patients in group B experienced complete neurosensory recovery. One year of follow-up was needed. The neurosensory response of all patients returned to the presurgical situation. The neurosensory response was 80-93% in the sixth month in group A, in which they had not undergone preoperative CBCT. In this present study, the amount of careful nerve manipulation intraoperatively based on the CBCT values played a crucial role in the neurosensory recovery of group B. A study conducted by Aizenbud D et al. [18] also found no significant NSI after BSSO. He decided that the fact that he used CBCT technology as part of his pre-surgery planning may have helped explain why his NSI results were so good.

One limitation of this study is that the sample size was relatively small. This present study does not reflect surgical factors such as the surgeon’s skill. The vertical and horizontal position of the lingula was not taken as a parameter due to the lack of proper surgical field of vision and the trial to find an anti-lingula may cause a higher amount of compression and stretching force on the neurovascular bundle, but the horizontal cut at the posterior region was done above the lingual.

## Conclusions

According to the findings of this study, a CBCT scan is a valuable and reliable modality in the preoperative evaluation of patients having BSSO, notably for the assessment and mapping of the mandibular canal. The CBCT, rather than panoramic radiography, is a crucial tool in the surgeon's arsenal because it is useful during the informed consent process as well as intra-operatively when it can aid in minimizing nerve injury during the BSSO surgery, as evidenced by higher sensation scores.
